# Cellular and molecular mechanisms that regulate mammalian digit tip regeneration

**DOI:** 10.1098/rsob.200194

**Published:** 2020-09-30

**Authors:** Mekayla A. Storer, Freda D. Miller

**Affiliations:** 1 Program in Neurosciences and Mental Health, Hospital for Sick Children, Toronto, Canada M5G 1L7; 2 Department of Molecular Genetics, University of Toronto, Toronto, Canada M5G 1A8; 3 Department of Physiology, University of Toronto, Toronto, Canada M5G 1A8; 4 Institute of Medical Sciences, University of Toronto, Toronto, Canada M5G 1A8

**Keywords:** regeneration, digit tip, blastema, wound healing, mammalian, mesenchymal precursors

## Abstract

Digit tip regeneration is one of the few examples of true multi-tissue regeneration in an adult mammal. The key step in this process is the formation of the blastema, a transient proliferating cell mass that generates the different cell types of the digit to replicate the original structure. Failure to form the blastema results in a lack of regeneration and has been postulated to be the reason why mammalian limbs cannot regrow following amputation. Understanding how the blastema forms and functions will help us to determine what is required for mammalian regeneration to occur and will provide insights into potential therapies for mammalian tissue regeneration and repair. This review summarizes the cellular and molecular mechanisms that influence murine blastema formation and govern digit tip regeneration.

## 1. Introduction

The ability of animals to regenerate lost body parts has fascinated scientists for centuries. In part, this is due to fact that regenerative ability between organisms varies so greatly. On one end of the spectrum, non-mammalian vertebrates, such as amphibians, display a remarkable capacity to heal in a scar-free manner and to regenerate appendages, even as adults [1,2]. For example, fish can regenerate their fins and heart [3–5], and urodele amphibians such as the Mexican axolotl and red-spotted newt can heal wounds perfectly and regenerate not only full limbs and appendages upon amputation but also the brain and spinal cord [6–11]. In contrast, the capacity for multi-tissue regeneration has largely been lost in mammals, raising the question of why this is so, since this ability has major implications for therapeutic approaches to tissue repair. There are, however, several exceptions to this rule, including skin regeneration in spiny mice [12], the closing of ear hole punches in rabbits and some rodents [13–16], the shedding and regrowth of deer antlers [17–19], and distal digit tip regeneration that occurs in rodents, monkeys and humans [20–25].

**Figure 1.**
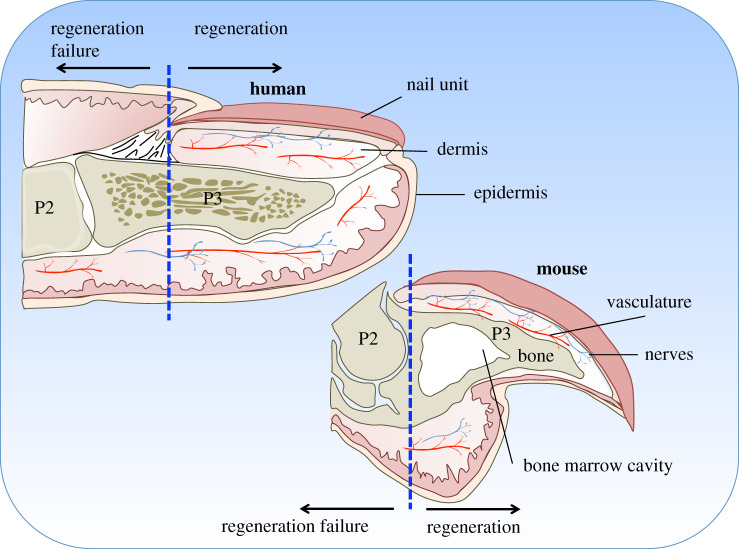
Anatomy of the human fingertip compared to the mouse digit tip. Both digit tips are similarly composed of a nail unit (dark pink), epidermis (orange), loose connective tissue dermis (light pink), vasculature (red), nerves (blue) and bone (brown). The second (P2) and third (P3) phalanges are indicated. Amputations removing the entire nail unit (indicated by the blue hatched line) result in regeneration failure.

Among the handful of mammalian regeneration models, the mouse exemplifies an ideal system to study spontaneous regeneration, as the process of murine digit tip regeneration is akin to, and clinically relevant to, human fingertip regeneration. Both digit tips are similarly composed of skin, nerves, blood vessels, bones and tendons (figure 1), and regeneration requires all of these tissues to regrow in a temporally and spatially precise manner to replace the original structure and restore functionality. Additionally, in both systems, digit tip regeneration is level dependent; injury following amputation of the distal region of the third phalangeal element regenerates, whereas amputations that occur more proximally, removing the nail bed, fail to regenerate [20–22,24–26]. This level-dependent regenerative ability provides a powerful model system for identifying mechanisms that endow regenerative capabilities in contrast to fibrotic healing.

While the clinical literature demonstrates that the human fingertip has regenerative ability if treated conservatively, it has been extensive studies in mice that have brought us closer to understanding the processes underlying this phenomenon. As seen in other species that regenerate, mouse digit tip regeneration is a multi-step process that first involves a slow wound-healing response and epidermal closure [21,22,27,28]. This is followed by the key step in digit tip regeneration, the genesis of the blastema; a transiently proliferating cell mass that generates the different cell types of the digit to replicate the original structure. How does the blastema form from mature tissues? What cell types are in the blastema and where do they come from? Is it a homogeneous population of pluripotent progenitors or a complex mixture of lineage restricted cells each already pre-determined to form a specific tissue? What are the molecular mechanisms that promote successful regeneration? These are some of the central questions of regenerative biology and are therefore the focus of this review. Accordingly, if we can understand how the blastema forms and functions during digit tip regeneration then this will provide clues as to how we can stimulate regeneration and repair of limbs and other tissues in mammals.

## 2. Overview of mammalian digit tip regeneration

### 2.1. Anatomy of the murine digit tip

While mammalian digit tip regeneration has been predominately studied in neonatal and juvenile mice, it also occurs in adults [21,26,27,29], albeit over a somewhat longer timeframe. The regeneration competent region (see schematic in figure 1) consists of a triangular shaped bone with its associated bone marrow cavity and a footpad containing sweat glands, myoepithelial and luminal secreting cells and associated neurons, located ventrally at the base of the bone. The distal bone is encased by a translucent nail organ, an epidermal structure that includes the nail bed and is highly vascularized. The proximal region of the distal phalanx (third phalangeal element, P3) articulates with the distal end of the second phalangeal element (P2), forming the P2/P3 joint. Amputations through this region, which remove the nail bed, elicit a non-regenerative response [21,22,26,30]. A thin layer of loose connective tissue containing fibroblasts, vasculature and nerves separate the distal bone from the nail epidermis.

### 2.2. The regenerative process—a brief overview

Mammalian digit tip regeneration is a multi-step process involving a wound healing response comprising inflammation, tissue histolysis and epidermal closure; formation of a transient blastema; and ultimately skeletal morphogenesis and re-differentiation to restore the amputated structures. Upon removal of the distal region of the digit tip, the injured epidermis retracts and attaches to the periosteal surface of the stump bone at a level proximal to the original amputation plane [27]. This is followed by an infiltration of monocytes to the wound site, which then differentiate into macrophages or undergo fusion to form multinucleated osteoclasts that degrade the bone and surrounding tissue [28,31]. The wound epidermis then closes through the newly eroded bone, resulting in a secondary amputation at a more proximal level, the expulsion of the distal bone fragment and the formation of the blastema [27]. Evidence suggests that there may be a direct relationship between the degree of bone degradation and the size of the blastema. When amputation wounds were induced to close prematurely using a cyanoacrylic wound dressing, bone histolysis was inhibited and a significantly smaller blastema was generated [28]. In contrast, enhancing the period of bone degradation experimentally through daily application of hyperbaric oxygen resulted in the formation of a larger blastema [32], which could be theoretically attributed to greater progenitor availability.

Blastema maturation is characterized by the onset of skeletal differentiation and progresses in a proximal to distal manner by intramembranous ossification [21,27]. Bone regeneration occurs rapidly with osteoblasts secreting osteoid that is subsequently converted to a bony matrix, forming woven bone. This replacement bone is characterized by numerous trabecular spaces that make it distinguishable from the stump. However with time, the bone increases in density and the trabecular spaces become smaller. Despite this remodelling, the bone of the regenerated digit tip is still histologically distinct [27,33]. Coincident with this bone regrowth, the loose connective dermal tissue and vasculature also regenerate and digit tip morphology and patterning is restored [27].

## 3. Cellular mechanisms of digit tip regeneration

### 3.1. The cells of the mammalian blastema—multipotentiality and origins

One of the fundamental challenges surrounding regenerative responses has been to define the source and potency of progenitor cells that form the blastema and contribute to the regenerated tissue. Historically, the blastema was presumed to be a collection of proliferative, homogeneous and by default pluripotent cells that give rise to the regenerated tissue [34]. This classical assessment of the nature of the blastema was based upon cell morphology in the regenerating newt limb [34]. However recent studies of digit tip regeneration using genetic lineage tracing and single cell transcriptomic profiling have demonstrated that the murine digit tip blastema is heterogeneous in nature and comprised of a variety of different cell types [29,35–39]. These cell types include endothelial cells and lymphatic endothelium, vascular smooth muscle cells, pericytes, Schwann cells, macrophages, neutrophils, T-cells, monocytes, pre-osteoclasts and various types of mesenchymal cells including osteoblasts [27,29,31,35,36,38,39]. Although this list of cell types is extensive, 80–85% of the blastema is comprised of mesenchymal cells that express the marker *Pdgfra* [29,39] and will be herein referred to as the mesenchymal blastema.

What happens to the mesenchymal blastema cells in the mammal? Is regeneration a simple scenario where bone regenerates bone and dermis regenerates dermis? Do blastema cells remember their origin and only differentiate into the cell types from which they arose? The answer appears to be no. Previous genetic lineage tracing studies in mice, using cell type specific reporter expression have demonstrated that the cells of the mammalian digit tip blastema are restricted in terms of their developmental lineage [35,36]. Epidermal cells were shown to only give rise to epidermis while endothelial cells only gave rise to endothelium, thereby remaining faithful to their tissue of origin in the regenerated tissue [35,36]. However, while these studies clearly demonstrate that the mammalian blastema is not pluripotent across developmental germ layers, the potential to create diverse cell types within germ layers was not fully investigated in these earlier studies. More recently, using genetic lineage tracing techniques in mice, Storer and colleagues [39] found that *Dmp1-*positive cells that reside in the bone under homeostatic conditions were able to switch cell fates within the mesenchymal lineage during regeneration, contributing to both dermis and bone in the regenerated digit tip. Similarly, when dermal fibroblasts were transplanted into a regenerative, but not non-regenerative digit, they were shown to acquire a blastema phenotype and contribute to bone regeneration [39]. These results indicate that cells within the mammalian digit tip blastema maintain some flexibility in terms of their fate and suggest that the local environment may, in part, specify the cell's ultimate contribution to the regenerated tissue. Indeed, a recent study using transgenic multicolour ‘brainbow’ axolotls and long-term imaging of digit tip regeneration concluded that in this organism the timing of dermal migration into the blastema is what biases the fate of these cells towards contribution to the skeleton or dermis [40]. Taken together, these data indicate that the mammalian blastema contains a mixture of both unipotent and multipotent progenitors, although further studies using cell-type-specific markers will be required to locate and trace these cells *in vivo*.

Where do the mesenchymal blastema cells come from? As was previously seen in amphibians [40,41], parabiosis studies indicate that they probably originate from local cells resident within the uninjured digit tip, since donor-derived cells from outside the digit tip made little or no contribution to regeneration in recipient mice [36]. It is also likely that they originate from local pre-existing mesenchymal cells since lineage-tracing studies showed that ectodermal and mesodermal-derived digit tip cells do not cross germ-line lineage boundaries during regeneration [35,36,42–44]. More definitive support for this conclusion comes from recent work showing that following distal digit tip amputation pre-existing *Pdgfra-*expressing mesenchymal cells from local tissues in the uninjured adult murine digits establish the regenerative blastema [39].

**Figure 2.**
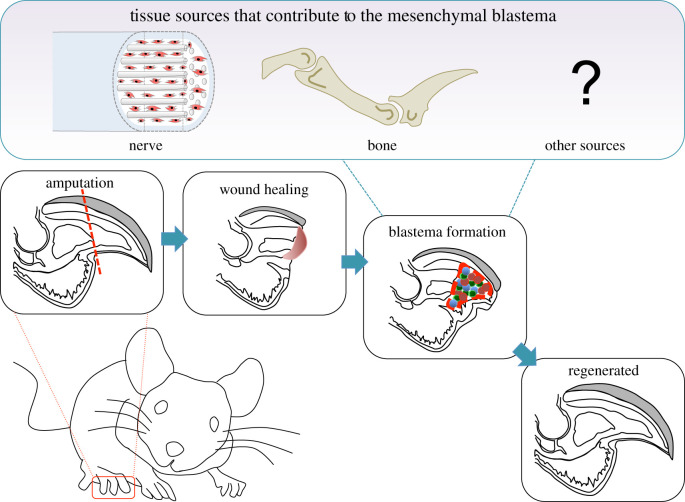
Mouse digit tip regeneration is a multi-stage process. Amputation of the distal third of the mammalian digit tip (indicated by the red hatched line in the left lower panel) results in a multi-stage regenerative process that includes epidermal closure and wound healing (second lower panel), blastema formation (third lower panel) and differentiation to form the regenerated digit tip (fourth lower panel). The known tissue sources that provide cells for the mammalian digit tip blastema, mesenchymal cells from the nerve (shown in red) and cells from the bone are shown in the top panel.

What then are the local mesenchymal tissues that contribute cells to the blastema? Resolving this question has been the goal of many researchers, but until recent years little progress has been made due to the lack of satisfactory molecular markers to lineage-trace specific cell populations. Nonetheless, it is now emerging that blastema cells in the murine digit tip derive from several parental tissues (figure 2). A recent study performed by Carr and colleagues [37] showed that a subpopulation of blastema cells are neural crest-derived mesenchymal cells that migrate out of local nerves following injury. Additionally, lineage tracing using *Dmp1*, which marks both osteoblasts and osteocytes, indicates that 26% of cells in the adult murine blastema originate from bone-associated cells [39]. These cells probably derive from the bone lining, which contains *Dmp1-*positive osteoblasts, since previous studies have demonstrated a requirement of the periosteum for successful digit tip regeneration [33,45]. What mesenchymal tissues generate the remaining mesenchymal blastema cells? These cells plausibly originate from the dermis of the loose connective tissue, nail bed mesenchyme and/or bone marrow stroma. In axolotl limb regeneration, there is evidence that dermal fibroblasts contribute disproportionally to the blastema and display a high level of plasticity when cellular contributions from other tissues are restricted [7,46,47]. In this regard, it is well established that regeneration does not occur following digit tip amputation proximal to the nail bed [22,26,48]. Thus it is tempting to speculate that the nail bed mesenchyme provides an abundant source of *Pdgfra-*expressing cells that are already in a ‘primed’ inductive state, and that perhaps they initiate and contribute robustly to the blastema.

### 3.2. Generating the source material

**Figure 3.**
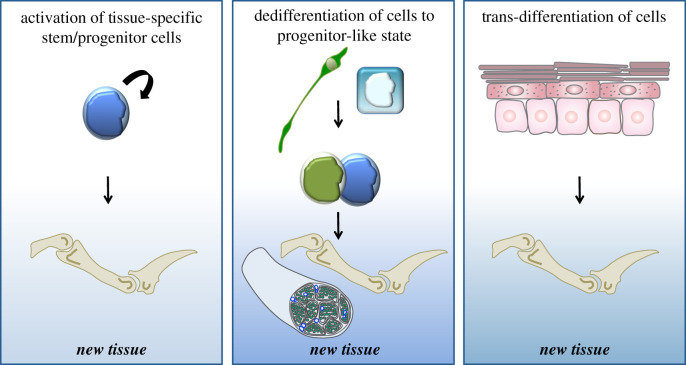
Potential mechanisms for producing new cells for regeneration. Left panel: activated stem or progenitor cells can self-renew and produce one or more differentiated cells that contribute to the regenerated tissue. Middle panel: dedifferentiation is the process by which mature cells lose their differentiated phenotype to produce a dividing precursor cell that can then produce more differentiated cells to contribute to the regenerated tissue. Right panel: trans-differentiation involves the direct change of one cell type into another, bypassing the progenitor state.

How do cells within uninjured local tissues contribute to formation of the blastema? There are three broad mechanisms that are commonly considered when answering this question (figure 3). First, new types of cells could be produced by activation of tissue resident stem cells. Indeed, this has been shown to be the case during axolotl limb regeneration and *Xenopus* tail regeneration where blastema formation involves satellite stem cell activation [49–53]. Second, new cells could be produced by reversion of the differentiated state to produce a dividing cell that acts as a progenitor cell—commonly referred to as dedifferentiation. Research in vertebrates has indicated that many of the blastema cells arise from dedifferentiation of the mature tissues that remain in the local vicinity following amputation [6,7,42,44,54–59]. For example, six studies using zebrafish as a model system, two in the heart and four in the fin, established that cardiomyocytes and osteoblast cells dedifferentiate and proliferate during heart and fin regeneration [42,44,56–59]. Finally, new cell types could arise as a result of trans-differentiation, the lineage conversion of a defined cell into another cell type, bypassing the progenitor state. While trans-differentiation has been described in amphibian appendage and zebrafish fin regeneration experiments in which certain tissues or cells were removed or irradiated, this mechanism is not thought to significantly contribute to the regenerated tissues [44,60–62].

Which of these mechanisms allow local tissues to contribute cells for the regenerating digit tip blastema? Several studies have started to address this question, but the answer is still not clear. In particular, lineage-tracing studies have examined the germ-line origin of the different cells that contribute to the regenerating digit tip [30,35,36,48], and provided evidence that epithelial and mesodermal digit tip cells do not trans-differentiate between germ-line boundaries during regeneration. Moreover, two recent transcriptomic analyses of adult murine blastema digit tip cells addressed this issue but reached somewhat distinct conclusions. In one study, blastema mesenchymal cells were found to be distinct from mesenchymal cells in the uninjured mesenchymal digit tip tissues [39] leading to the conclusion that the uninjured cells must somehow acquire a blastema phenotype during regeneration, more consistent with ideas about dedifferentiation. The second study instead found some transcriptionally similar mesenchymal cells in both the uninjured and regenerating digit tips, and concluded that resident stem cell populations in the uninjured tissues were responsible for blastema formation [38]. It is likely that both models are true to some degree, but the precise contribution of the two proposed mechanisms will await additional experimentation.

### 3.3. Forging a new path—regeneration versus development

Fundamentally, regeneration shares many similarities with development, including the necessity for cell communication, cell differentiation, morphogenesis and tissue patterning. Indeed, at the molecular level, the expression and function of many developmental genes are common to both processes and it is generally accepted that developmental mechanisms govern the later stages of the mammalian digit tip regeneration response. However, despite these commonalities, there are some distinct differences between these two processes and this raises the question of the extent to which regeneration of the mammalian digit tip recapitulates development. In particular, during early regeneration, do cells of the blastema simply revert back to an embryonic like state, which enables them to regenerate the missing appendages? To gain insight into this question, two independent research groups have used single cell global transcriptomic approaches to compare the transcriptional profile of the blastema to embryonic and early postnatal animals at key stages in development, in two different regenerative species. First, Gerber and colleagues [63] demonstrated that during axolotl limb regeneration, mature connective tissue in the uninjured adult limb reverts to a relatively homogeneous progenitor state that recapitulates an embryonic limb bud-like phenotype. In contrast to the axolotl paradigm, Storer and colleagues [39] determined that during murine digit tip regeneration, the cells of the mammalian mesenchymal blastema do not reiterate development, but instead follow a distinct adult regenerative transcriptional trajectory that nonetheless includes expression of many developmentally important genes. These different findings reinforce the concept that regenerative mechanisms are not universal and highlight the importance of studying regeneration in mammalian species, despite their lower regenerative capacity.

A second significant difference between mammalian digit tip development and regeneration occurs during bone formation. During embryonic development all of the bones in the developing limb, including the distal digit, form by the process of endochondral ossification. During this process chondrocytes undergo hypertrophy to establish a cartilage template that is eventually replaced by new bone [64]. In contrast, during mammalian digit tip regeneration new bone is generated through the process of intramembranous ossification in which osteoblasts condense directly to differentiate bone [21,27]. Notably, bone formation during regeneration is imprecise, resulting in digits with a bone volume that is significantly larger than unamputated controls [27]. However, while distinct from development, this type of intramembranous ossification is also seen during adult mammalian bone healing, consistent with the idea that digit tip regeneration follows a distinct adult trajectory that is a combination of adult tissue repair and developmental limb formation mechanisms.

## 4. Molecular mechanisms of digit tip regeneration

### 4.1. Establishing a signalling centre—the role of the nail organ

**Figure 4.**
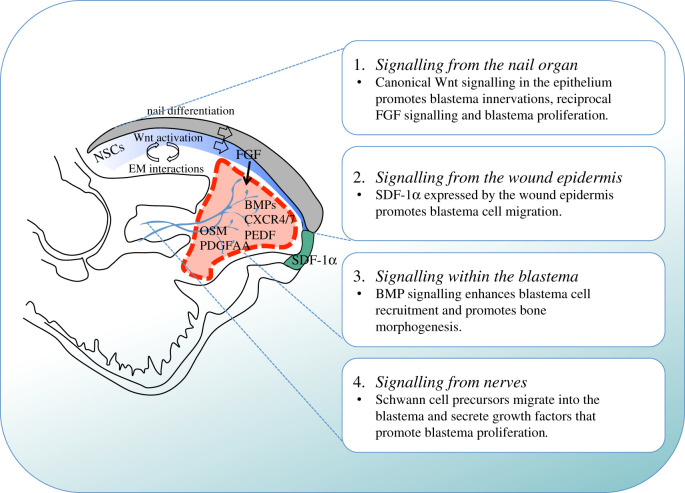
Known signalling mechanisms that contribute to mammalian digit tip regeneration. Indicated are the signalling mechanisms that originate from the nail organ (1), from the wound epidermis (2), within the blastema (3) and from nerves (4). During distal digit tip amputation the wound site is covered by regenerating nail epithelial cells that arise from the nail stem cells (NSCs). This epithelium activates Wnt signalling that promotes differentiation of NSCs that give rise to the nail plate. Additionally, through epithelial–mesenchymal (EM) interactions, this Wnt signalling promotes blastema innervation, which in turn enables FGF2 expression in the nail epithelium and promotes blastema cell proliferation. Blastema cell migration is stimulated by the cytokine SDF-1α that is expressed by the wound epidermis while the reciprocal receptors CXCR4/7 are detected on blastema cells, together with BMPs and the anti-angiogenic factor *Pedf*. Schwann cell precursors that arise from injured peripheral nerves migrate into the blastema and secrete growth factors including oncostatin M (OSM) and platelet derived growth factor AA (PDGFAA) that promote blastema proliferation. Nerves are shown in blue; the wound epidermis is indicated in green; the blastema is indicated by the red-hatched line; the nail epithelium is indicated in blue and the nail plate is coloured grey.

In mammals, successful regeneration of the digit tip is dependent on the level of amputation. Removal of the distal portion of the terminal phalanx results in near perfect restoration of the original structure [20,24,25], while amputations past the proximal insertion point of the nail fail to regenerate and culminate in scar formation [21,26,27,65]. These observations have led to the conclusion that the presence of the nail at the amputation plane is necessary to mount a regenerative response. Why is this so? One idea is that the nail, which is epithelial in nature, is a source of multipotent digit-specific progenitors essential for the regenerative process. However, lineage-tracing studies have demonstrated that during digit tip regeneration epidermally derived cells contribute exclusively to the newly formed epidermis [35,36]. Alternatively, perhaps the nail organ provides a molecular signalling centre that is necessary for its continuous growth and after injury this signalling centre becomes essential for regeneration. Several lines of research support this latter hypothesis. First, an elegant study conducted by Takeo and colleagues [30] demonstrated that canonical Wnt signalling in the nail epidermis is necessary for nail stem cell differentiation under homeostatic conditions and is equally important for the mesenchymal response during regeneration. In particular, conditional deletion of β-catenin from the epithelium following digit tip amputation impaired nail regeneration, disrupted FGF signalling necessary for nerve recruitment and blastema formation, and inhibited BMP signalling, which is essential for the generation of new bone [30]. Second, LGR6, an agonist of Wnt signalling, has been found to be a specific marker for nail stem cells within the nail matrix, and its activity is ultimately necessary for digit tip regeneration [66]. Third, transplantation of the nail organ to the site of non-regenerative digit tip amputations resulted in ectopic bone growth rather than the normal fibrotic wound-healing response [67]. These findings highlight the importance of epithelial to mesenchymal interactions in the regenerative response and demonstrate that both Wnt signalling and the nail organ dictate the level dependency of mammalian digit tip regeneration (figure 4).

### 4.2. Signalling from the wound epidermis

The wound epidermis has long been recognized to play an integral role in promoting successful regenerative responses. In particular, classical experiments in salamanders showed that the injured epithelium undergoes a nerve-dependent transition into a specialized structure called the apical ectodermal cap, which functions as a signalling centre to maintain the underlying mesenchyme in an undifferentiated, proliferative state [68–71]. These findings raise the intriguing possibility that the wound epidermis may also be an important signalling centre for mammalian digit tip regeneration. Support for this concept comes from the observation that soon after completing wound closure, the wound site undergoes a striking transformation to generate the digit blastema [27]. However, little is known about why this is so, although a study from Lee and colleagues [72] provides some insight into this issue. In particular, this study showed that the cytokine stromal derived factor 1α (SDF-1α) is expressed by the wound epidermis and by a sub-population of endothelial cells, and that the blastema cells express the two SDF-1α receptors, CXCR4 and CXCR7. When mice were treated with AMD3100, a CXCR4 specific antagonist, there were deficits in blastema cell migration and partial inhibition of regenerative bone growth. Moreover, ectopic expression of SDF-1α by cells engrafted at the site of non-regenerative amputations induced local cell migration and a partial regenerative response. Thus, SDF-1a, secreted in part by the wound epidermis, is essential for the early stages of the regenerative response (figure 4). These results are the first to hint at an important role for the wound epidermis during mammalian digit tip regeneration and lay the groundwork for future studies into this intriguing topic.

### 4.3. Blastema formation and maturation

The mechanisms underlying initiation, formation and maintenance of the mammalian digit tip blastema are still not well understood, although several intrinsic and extrinsic cues have been identified. Since the nail is essential for digit tip regeneration, one approach has been to examine genes that are expressed in this region such as the transcriptional repressors *Msx1* and *Msx2* [73]. Using mice carrying targeted deletions of *Msx1* or *Msx2*, Han and colleagues [74] showed that fetal mice deficient in *Msx1* did not readily regenerate amputated digit tips and that this deficit could be restored by exogenous application of BMP4. However, *Msx2* and *Dlx5*, homeobox transcription factors that are also expressed in the regenerating fetal digit tip blastema, were not found to be essential for regeneration either when deleted alone or in combination [75]. Notably, limb development is normal in *Msx1-*deficient mice, supporting the conclusion that *Msx1* functions in a regeneration specific manner. Indeed, even human *in vitro* fetal digit tip amputation models report expression of *Msx1* in the developing and regenerating digit tip [76]. Nonetheless, while these findings hold true for embryonic digit tip regeneration, *Msx1* was not found to be expressed in the blastema of neonatal mice [35,74]. Moreover, while it is expressed in regenerating adult blastema mesenchymal cells, it was not found to be enriched relative to mesenchymal cells from regeneration-incompetent amputations [39]. In summary, while *Msx1* has an important role during fetal digit tip regeneration, its role in postnatal or adult regeneration has yet to be functionally assessed.

In contrast to *Msx1*, it has been well documented that BMP signalling is required for digit tip regeneration irrespective of developmental age. Specifically, BMP2 has been demonstrated to enhance cell recruitment to the blastema by activating the SDF-1/CXCR4 signalling pathway [72]. BMP4 is expressed by cells of the blastema [21] and when BMP signalling is inhibited using the BMP antagonist, *Noggin*, digit tip regeneration fails [74,77]. Finally, mice with conditional deletion of β-catenin from the epithelium present with deficits in regeneration and were subsequently shown to lack *Bmp4* expression in the digit [30]. Taken together, it is clear that BMP signalling is one of the integral pathways necessary for successful mammalian digit tip regeneration.

While this handful of studies indicate the importance of BMP signalling for blastema formation and skeletal regeneration (figure 4), it is the research treating proximal regeneration-incompetent amputations with BMP molecules that highlights their potential therapeutic importance. Specifically, treatment of non-regenerative amputations with either BMP2 or BMP7 induced longitudinal regrowth of the middle phalanx [45,77,78]. Notably, bone regrowth in these experiments occurred by endochondral ossification, implying that BMP-induced regeneration recapitulates digit tip development rather than adult digit tip regeneration [45,77,78]. In addition, a recent study demonstrated that sequential treatment with BMP2 followed by BMP9 stimulates regeneration of the bone and joint by cells that would otherwise undergo fibrotic healing [79]. This body of work thus leads to the important conclusion that proximal amputation wound cells possess regenerative potential but lack the necessary environmental cues for inducing a successful regenerative response.

How then is the microenvironment within the blastema controlled? Several studies indicate that vascularization plays a key role. Specifically, one study showed that dynamic changes in oxygen tension are critical for regulating phase transitions important for the regenerative response [80]. During the initial stages of murine blastema formation, the blastema has been shown to be specifically hypoxic yet it remains avascular and many of the blastema cells express the anti-angiogenic factor, *Pedf* [27,80,81]. Addition of exogenous VEGF or BMP9, which functions upstream of *Vegfa*, to the regenerating digit tip was found to induce precocious angiogenesis within the blastema and inhibit digit tip regeneration [81]. Additionally, BMP9-induced inhibition of regeneration could be rescued by treating the digits with exogenous PEDF [81]. These studies also noted that the initiation of osteogenesis that occurs when blastema cells start to differentiate coincides with the induction of *Vegfa* expression [80,81]. Collectively, these results indicate that angiogenesis is tightly regulated during mammalian digit tip regeneration and plays an important role in coordinating blastema formation and maturation.

### 4.4. The role of innervation

Many regenerative processes rely on the presence of intact peripheral nerves. This is best described in amphibians where surgical denervation prior to limb amputation results in the inhibition of blastema formation, and ultimately leads to regeneration failure [82–84]. In these instances, peripheral nerves are thought to stimulate regeneration, at least in part, through the secretion of growth factors that positively influence the local microenvironment such as FGFs [85], BMPs [86], anterior gradient protein [8] and neuregulin 1 [87]. Similar to amphibians, mouse digit tip regeneration is a peripheral nerve-dependent event where surgical denervation prior to digit tip amputation resulted in a delayed wound healing response, attenuated blastema growth and reduced bone regrowth [29,30,88,89]. These observations thus raise the question as to what nerves contribute and how they exert their pro-regenerative effects in mammalian digit tips.

To address the contribution of peripheral nerves to mammalian digit tip regeneration, Johnston and colleagues [29] used genetic lineage tracing techniques to demonstrate that nerve-associated *Sox2*-positive dedifferentiated Schwann cells (termed SCPs) are present within the regenerating digit tip blastema but absent following denervation. Loss of function experiments established that when SCPs were dysregulated or ablated, mesenchymal precursor proliferation in the blastema was decreased and bone regeneration was impaired. Conversely, regeneration could be rescued by injecting cultured SCPs into the amputated digits of denervated or Schwann cell-depleted mice [29]. While these genetic experiments demonstrated a novel role for SCPs in digit tip regeneration, several question remained; why are these cells necessary for this process and what are they doing at the molecular level? One explanation is that SCPs located within the blastema function to secrete factors important for the regenerative response. To test this idea, the authors used transcriptomic and proteomic analysis to model paracrine networks between SCPs and the mesenchymal cells of the blastema. Of the predicted SCP-derived factors, platelet derived growth factor AA (PDGF-AA) and oncostatin M (OSM) were selected for verification and shown to stimulate proliferation of mesenchymal precursor cells and to rescue the digit tip regeneration deficits that occur following denervation (figure 4). Notably, a subsequent study showed that Schwann cells also promote murine mandibular repair by a very similar paracrine mechanism [90]. Ultimately, this work addresses the mechanisms by which SCPs contribute to mammalian digit tip regeneration and has provided the digit tip regeneration community with numerous newly identified ligand–receptor pairs for further study.

In addition to providing neurotrophic factors that promote mammalian tissue regeneration nerves have also been shown to contribute to the regenerative process through a second mechanism. In a recent study, Carr and colleagues [37] demonstrated that injured peripheral nerves provide a reservoir of mesenchymal precursor cells that can directly contribute to mesenchymal tissue repair and regeneration. Using a combination of single cell transcriptomic approaches and lineage tracing, they identified a distinct population of mesenchymal neural crest-derived cells with characteristics of mesenchymal precursor cells. These neural crest-derived mesenchymal cells were found in nerve fascicles, in close apposition to Schwann cells and axons. When differentiated in culture they generated cells with characteristics of adipocytes, bone and cartilage cells and when transplanted into mice with bone or skin injuries, they migrated out of the nerve and into the damaged bone and dermis, respectively, to contribute to healing. Notably, these neural crest-derived nerve mesenchymal cells were also found to directly contribute to the mammalian blastema and to ultimately contribute to the newly regenerated bone and dermis in the digit tip [37]. In summary, this work has addressed the question of nerve dependency during mammalian digit tip regeneration and demonstrated that nerves contribute to regeneration through two complementary mechanisms: first, the provision of dedifferentiated Schwann cells that secrete growth factors into the local microenvironment; and second, through the contribution of nerve-derived mesenchymal cells that are able to differentiate into the regenerated mesenchymal tissues in response to local cues.

## 5. Concluding remarks

The blastema is the critical structure separating a successful regenerative response from regenerative failure. Historically, much of what we know about the mechanisms of blastema formation and appendage regeneration has come from studying animals that display enhanced regenerative abilities. However, studies focused on mammalian models are also necessary for understanding why mammals do not regenerate well, and for developing potential new therapies for regeneration and tissue repair. In this regard, the adult mammalian digit tip provides an ideal model system since amputation at a distal level that includes the nail results in regeneration while amputation at a somewhat more proximal level leads to a fibrotic response. Various studies taking advantage of this system have shown that the mammalian blastema is a heterogeneous population of cells that arise from a variety of local parental tissues in the digit tip including cells from the nerve and bone, much like amphibians. However, by contrast to amphibians, mammalian digit tip regeneration represents a specifically adult regenerative response that does not simply recapitulate the developmental programme, but involves aspects of both development and adult tissue repair mechanisms. In spite of this progress, there are more questions that remain than have been answered. How is blastema formation initiated? Is it the wound epithelium and/or the invading immune cells that are important? Why is the nail necessary for appropriate regeneration? Is it important as a specialized signalling centre and/or does it provide a source of cells that are already primed for regeneration? Do cells in the region of non-regenerative amputations have full regenerative potential and, if so, what are the environmental cues that are necessary to unmask this potential? How important are patterning genes for appropriate regeneration? Are the mechanisms that underlie successful digit tip regeneration similar to mechanisms involved in tissue repair? And if so, can we harness these mechanisms in a general way to promote adult tissue repair? While this long list of questions is at first glance somewhat daunting, it also reflects a real opportunity to understand mammalian regeneration and repair using the many tools and technologies that we now have available, and to ultimately enhance these processes for therapeutic purposes in humans.

## Data accessibility

This article has no additional data.
